# SIMS of Organic Materials—Interface Location in Argon Gas Cluster Depth Profiles Using Negative Secondary Ions

**DOI:** 10.1007/s13361-018-1905-2

**Published:** 2018-02-21

**Authors:** R. Havelund, M. P. Seah, M. Tiddia, I. S. Gilmore

**Affiliations:** 1 National Physical Laboratory, Teddington, Middlesex, TW11 0LW UK; 2 Universita degli Studi di Cagliari, Dipartimento di Fisica S. P. Monserrato, Sestu Km 0.700, 09042 Monserrato, CA Italy

**Keywords:** Matrix effects, Secondary ion mass spectrometry, Depth profiling, Gas cluster ion beam, Quantification, Interface location, TOF-SIMS, SIMS, GCIB

## Abstract

A procedure has been established to define the interface position in depth profiles accurately when using secondary ion mass spectrometry and the negative secondary ions. The interface position varies strongly with the extent of the matrix effect and so depends on the secondary ion measured. Intensity profiles have been measured at both fluorenylmethyloxycarbonyl-l-pentafluorophenylalanine (FMOC) to Irganox 1010 and Irganox 1010 to FMOC interfaces for many secondary ions. These profiles show separations of the two interfaces that vary over some 10 nm depending on the secondary ion selected. The shapes of these profiles are strongly governed by matrix effects, slightly weakened by a long wavelength roughening. The matrix effects are separately measured using homogeneous, known mixtures of these two materials. Removal of the matrix and roughening effects give consistent compositional profiles for all ions that are described by an integrated exponentially modified Gaussian (EMG) profile. Use of a simple integrated Gaussian may lead to significant errors. The average interface positions in the compositional profiles are determined to standard uncertainties of 0.19 and 0.14 nm, respectively, using the integrated EMG function. Alternatively, and more simply, it is shown that interface positions and profiles may be deduced from data for several secondary ions with measured matrix factors by simply extrapolating the result to *Ξ* = 0. Care must be taken in quoting interface resolutions since those measured for predominantly Gaussian interfaces with *Ξ* above or below zero, without correction, appear significantly better than the true resolution.

**Electronic supplementary material:**

The online version of this article (10.1007/s13361-018-1905-2) contains supplementary material, which is available to authorized users.

**Graphical Abstract:**
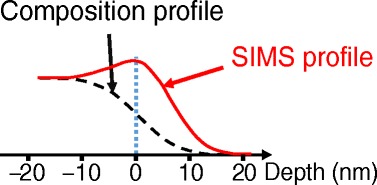
ᅟ

## Introduction

The location of interfaces in sputter depth profiling with surface analysis has been an important issue since the first depth profile experiments in the 1970s. The early effort was for metallic and inorganic material layers and searched for ways to improve the depth resolution. This was important, for example for assessing diffusion, segregation, restructuring or contamination at buried interfaces which may lead to device failure. The better the depth resolution, the more accurately separate layers are resolved and the more accurately the interface is located. In secondary ion mass spectrometry (SIMS), this led to efforts by Dowsett, Wittmaack and many others to reduce the primary ion beam energy. For example, in the study of boron delta layers in silicon, Chu and Dowsett [1] show that the component terms of the depth resolution reduce with the O_2_^+^ primary ion beam energy right down to 250 eV. Low energies were vital and led to the development of new low energy ion guns. The profiles were described by the function often known as a Dowsett function [1–3] where the position of the delta layer is given by Dowsett et al. [4] as the centroid of the compositional profile. They note, however, that the true delta position should be associated only with the sample-related part of the response function. The profile comprised a leading edge exponential, a trailing edge exponential and a Gaussian center, all convolved with each other. This was given in a simple analytical formula. Littmark and Hofer [5] show that the centroid is correct except near the surface where the centroid may be too deep. This is reasonable since the measured profile for the surface monolayer is an exponential decay [6] arising from the statistics of sputtering in that layer. At greater depth, ion implantation and recoil mixing move the centroid shallower and deeper, respectively, until equilibrium is attained.

Wittmaack et al. [7] in 2001 questioned if the peak or the centroid, in the SIMS measured profile for a delta layer, is the better marker. They showed that the peak was more affected by the beam energy than the centroid and attributed this to the changes in sputtering rate during the initial transient effect at the surface. At depths beyond the initial transient, the centroid of delta functions can mark positions extremely accurately and Seah et al. [8] show data for organic materials in this way with very low scatters of 0.4 nm in depth over 250 nm. An example of the rate change in the outermost 5 nm of organic material is given by Seah et al. [9] who point out the need to consider whether the interface is blurred locally (so that the composition changes slowly) or if it is sharp locally and blurred by the summing of separate events over the wavy surface. This affects the calculation that applies the matrix terms to convert composition to secondary ion intensity and so, unfortunately, affects the finally deduced interface positions. In practice, the result will be somewhere between these two extremes.

In the delta layer studies in silicon using SIMS, the compositions are all sufficiently dilute that the matrix effects may be ignored and the amount of substance is proportional to the measured signal. In profiles using Auger electron spectroscopy (AES) or X-ray photoelectron spectroscopy (XPS), the matrix effects are usually weaker and these methods have been used to study many interfaces between materials. For AES and XPS, the information is integrated over some depth and this is allowed for by convolving, numerically, the exponential escape depth with typically an exponential sputtering effect function and an integral Gaussian function [10]. The computed result is compared with the measured data and iteratively optimized. Hofmann [11], in his Mixing-Roughness-Information Depth (MRI) model associates the three above convolving parameters with the information depth, the atomic mixing and the roughness in the sample. This model has proved effective over many years in AES and XPS studies.

The identification of the locus of the interface in depth profiles is important since this is used to characterize samples and also, importantly, to define sputtering rates and layer thicknesses. There will, of course, be changes in the sputtering rate through interfaces [12], although that issue is largely ignored. Kim et al., in their SIMS studies of Co and Pt layers [13], as well as Si and Ge layers [14], use a simple mathematical procedure claimed to renormalize the strong matrix effects in order to deduce the compositional profile. Then, despite the resulting profile asymmetry, they define the interface at the position where the composition has reached to 50% of the plateau values. In the Si/Ge system, Morris et al. [15] define the position for the interface as the center of mass of the function convolved with the step edge to describe the composition. This center of mass of the resolution function seems better for these asymmetric profiles since, in the absence of sputter yield changes, it will give the equivalent dose required to remove all the overlayer atoms. It is important to note that the relevant resolution function only includes, as Dowsett et al. [4] note, sample-related parts. For XPS, one would not include the electron information depth contribution—that simply allows one to see the interface earlier to an extent dependent on the energy of the characteristic electron selected. So, in XPS and AES, the leading edge exponential is not part of the relevant resolution function. In SIMS, the much shorter leading edge exponential appears to be part of the sputtering process and so is included. This is not anomalous since that same term should also be included for AES and XPS but is swamped by the electron escape term.

Many of the above analyses generally use monatomic or diatomic sputtering ions which permit high-quality depth profiles to be obtained from inorganic materials but entirely degrades the molecular structures in organic materials. The emergence of gentler sputter ion sources, generating large cluster sputtering ions, has expanded the applicability of sputter depth profiling to organic materials [16]. Using gas cluster ion beams (GCIBs), with typically more than 1000 atoms in the cluster, molecular depth profiles with minimal signs of ion beam damage can be obtained to depths of several micrometres [17] with depth resolutions as good as 5 nm [18]. Such organic depth profiling and 3D SIMS imaging using Ar GCIB sputtering have found a wide range of applications, for example for the analysis of organic electronic materials [19], polymers [17], tissue sections [20, 21] and cells [22]. Profiling with XPS, in general, does not provide the required specificity with organic materials.

Niehuis et al. [18], for depth resolution, adopt the 16 to 84% criterion for two sigma in an integral Gaussian profile matched to the measured intensity. They conclude, as in the earlier work, that low energy argon cluster ions give the sharpest profiles. A detailed analysis of many profile resolutions using argon gas clusters in SIMS is reported by Seah et al. [23]. These show full widths at half maximum for delta layers in the range 5 to 16 nm with the best results at low energy and high cluster size. Shard et al. [24] show, in addition to low energies for the sputtering Ar GCIB, low energies for the analysis beam are important to obtain the highest depth resolutions. Additionally, sample rotation can help, especially with layered samples rather than samples in which imaging is important [24, 25]. In principle, all the issues raised for monatomic and diatomic sputtering ions with inorganic materials will occur for GCIBs sputtering organic materials although there is often the added complexity of using a second, analysing, ion source for the SIMS part. As shown by Shard et al. [26], the profiles of delta functions are described by Dowsett’s function [1–3] although that function can be used to describe the topography developed by the cluster ion sputtering. Again, Seah et al. [27] show that the exponential tail, attributed to atomic mixing in inorganic studies, may arise from simple re-deposition of some of the larger organic fragments where an extremity remains entangled beyond the zone liberated. So, although the issues are similar in their behavior, the physical sources of the effects may be very different. Very important, indeed, is the matrix effect.

Shard et al. [28] developed a description of the matrix effect in organic materials and provided measurements of the effect for a selection of secondary ions arising from intimate mixtures of two molecular materials at three different compositions. They summarized the effect in the matrix term, *Ξ*, which will be introduced in the “Theory” section below. Recently, Seah et al. [29] showed that the equivalent thickness of a nominally 3-nm thick layer of fmoc-pentafluoro-l-phenylalanine situated between layers of Irganox 1010 could be consistently deduced as 3.22 ± 0.07 nm from the measured profiles for the different characteristic secondary ions if their matrix effects, measured as described by Shard et al. [28], had been removed. If the matrix effects are unknown, the layer thicknesses, as deduced from those secondary ions, spanned the range from 2 to 28 nm [29]; the larger measured thickness values being the result of strong matrix enhancement. In the present study, we build on these studies to evaluate the effective location of interfaces. This is critical since there is no alternative general method for depth profiling, with nanometre range depth resolution, organic films.

## Experimental

The experimental details are described by Shard et al. [30] and are detailed further in Havelund et al. [27]. Briefly, the sample was made partly to study the sputtering of uniform mixtures to establish matrix effects but contained the three pure layers at the surface used here: an outermost 100 nm of FMOC (fluorenylmethyloxycarbonyl-L-pentafluorophenylalanine, C_24_H_16_F_5_NO_4_, *M*_r_ = 477.4) followed by 100 nm of Irganox 1010 (pentaerythritol tetrakis(3-(3,5-di-tert-butyl-4-hydroxyphenyl)propionate), C_73_H_108_O_12_, *M*_r_ = 1177.6) and a further 100 nm of FMOC. The two materials, from Sigma-Aldrich, were each sublimed in a Qbox450 (Mantis Deposition Ltd. Thame, UK) with relevant monitoring, shuttering and sample rotation to create the layer structures as shown in Figure 1 of Shard et al. [30]. The evaporators were controlled by quartz crystal oscillators (QCOs) calibrated to relate their outputs to the thicknesses of each material deposited on the wafer substrates by ellipsometry using an M2000DI spectroscopic ellipsometer (Woollam, NE, USA).

**Figure 1:**
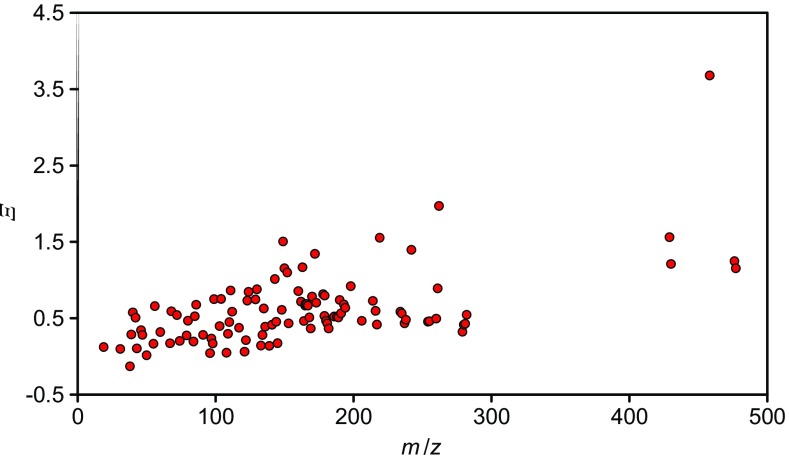
*Ξ* values for many negative secondary ions determined from the mixtures of FMOC and Irganox 1010 as a function of *m*/*z*

The above multilayer samples were depth profiled by SIMS using 5 keV Ar_2300_^+^ gas cluster primary ions in an ION-TOF TOF-SIMS IV instrument (ION-TOF GmbH) with the incident ions at 45° to the surface normal. The information-rich negative secondary ions were measured using 25 keV Bi_3_^+^ ions also at 45° incidence angle, but in an azimuth at 90° to the argon gas cluster sputtering beam. The sputtering beam was rastered, in interlaced mode, over an area of 500 μm × 500 μm and the analysis was in a central zone of 200 μm × 200 μm. The relative Bi_3_^+^ dose was <0.2% of the GCIB dose. Electron flood charge compensation using a 20 eV electron beam at 5 μA was used and the spectra were dead time corrected.

For measuring the matrix terms, samples with mixed layers of 0, 20, 50, 80, and 100% by volume were studied.

## Theory

The measured intensity allowing for the enhancement of the intensity of a secondary ion from A arising from a content of B, *ϕ*_B_, has been given by Shard et al. [28]. As before [12], we modify their equation to give

1 $$ \frac{I_{\mathrm{A}}/y}{I_{\mathrm{A}}^{\infty }/{y}_{\mathrm{A}}}\kern0.36em ={\phi}_{\mathrm{A}}+{\phi}_{\mathrm{B}}\kern0.24em \alpha\;\left[1-\exp\;\left(-\beta\;{\phi}_{\mathrm{A}}\right)\right] $$

where *I*_A_ is the characteristic ion intensity from A in the mixture and ${I}_{\mathrm{A}}^{\infty }$ is its intensity from pure A. The parameters *α* and *β* are the enhancement parameters, *y* is the yield volume for the mixture and *y*_A_ that for pure A. Since the signals are generated by 25 keV Bi_3_^+^ ions, the *y* yield volumes are for that primary ion. It is assumed that *I*_A_ is zero in material B and the volume fractions *ϕ*_A_ and *ϕ*_B_ sum to unity. Equation 1 differs from that by Shard et al. [28] by the inclusion of the sputtering yields for the analytical ion. The intensity for suppressed signals is

2 $$ \frac{I_{\mathrm{A}}/y}{I_{\mathrm{A}}^{\infty }/{y}_{\mathrm{A}}}\kern0.36em =\kern0.36em {\phi}_{\mathrm{A}}\kern0.24em \left\{1+\alpha\;\left[1-\exp\;\left(-\beta\;{\phi}_{\mathrm{B}}\right)\;\right]\;\right\} $$

i.e. Eq. 1 with *ϕ*_A_ and *ϕ*_B_ partly reversed since the charge transfer is in the reverse direction. Equation 2, here, differs from the equivalent equation by Shard et al. [28] by including the yields but also changes the sign of *α* so that *α* is positive for enhancement and negative for suppression. Seah et al. [12] show that, for *ϕ*_A_ < 0.8, *y*/*y*_A_ is effectively unity in this material system.

Fitting Eqs. 1 and 2 to the intensities for the samples with *ϕ*_A_ = 0, 0.2, 0.5, 0.8 and 1.0 gives values of *α* and *β* from which the summary parameter *Ξ* is determined. As before [29], we fit *α* and *β* and find that, approximately, for enhancement, *α* = [1 − exp(−*β*)]*P* + *Qβ* and, for suppression, *α* = exp(−*β*) − 1*.* This removes any unwanted correlation in fitting *α* and *β*. Thus, for each secondary ion, we fit just *β* with single values of *P* and of *Q* for all ions to establish *α*. This leads to the same quality of overall fitting as for the separate values of *α* and *β* each time. As before [29], we use *P* = 0.32 and *Q* = 0.17. The overall matrix enhancement factor, *Ξ*, is given by [28].

3 $$ \varXi \kern0.36em =\alpha \left(1-\left(2/\beta \right)+2\left\{\kern0.1em \left[1\hbox{-} \exp\;\left(-\beta \right)\right]/\left({\beta}^2\right)\right\}\right) $$

Secondary ions that are enhanced in the mixture have positive *Ξ* values. The intensity of a secondary ion with a *Ξ* value of 0, i.e. no enhancement, is linearly correlated with the sample composition. The matrix effect parameters, *α*, *β* and *Ξ* are specific to the individual secondary ion and the mixture of A with B. Example fits of the data for 5 secondary ions with *Ξ* values in the range 0 to 2 are shown in Online Resource 1, Figure S.1. It is clear that the data are well fitted by the equations above and that ions with *Ξ* > 1 show intensities for intermediate volume fractions of the mixture that are higher than the intensity from pure FMOC. We have measured the *α* and *β* values that govern the enhancement for 106 negative secondary ions from FMOC as described by Seah et al. [12] and these lead to the summary *Ξ* enhancement factor [29] given in Figure 1. We focus here on the negative secondary ions since they give the most detailed information for these materials. It is not expected that there will be any significant difference in the principles behind the behaviors in regard to the present study. Measurements comparing positive and negative secondary ions are the subject of a further study indicating that they may be stronger or weaker.

## Results and Discussion

### The Profiles

**Figure 2:**
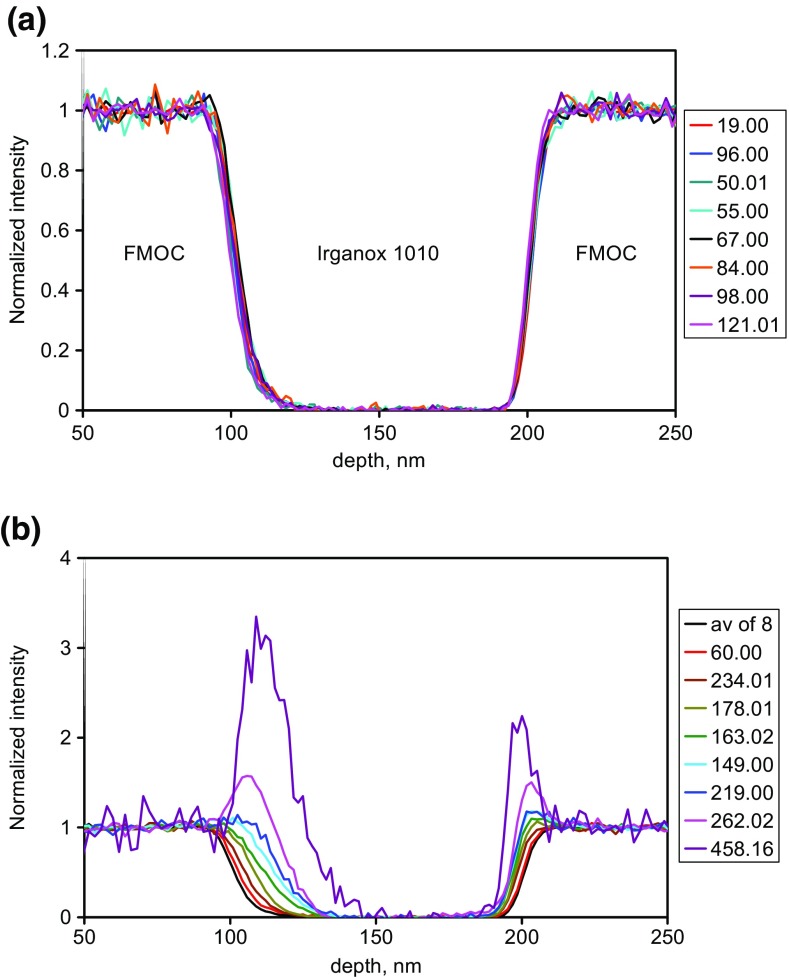
Normalized profiles for selected FMOC secondary ions with the depth scale calibrated to the interface positions using the varying sputtering rate, (**a**) ions with 0 < *Ξ* < 0.2 and (**b**) the average for the ions in (**a**) together with eight ions with *Ξ* up to the maximum value observed. The boxes to the right identify the secondary ion masses in *m*/*z*. The *Ξ* values of the ions in (**a**) from the top of the box to the right are 0.12, 0.04, 0.02, 0.16, 0.17, 0.20, 0.17 and 0.06 and in (**b**) are 0.12 for the average of the 8 in (**a**) followed by 0.32, 0.59, 0.82, 1.17, 1.51, 1.55, 1.97 and 3.68

Example depth profiles in this material sequence, as a function of the sputtering time are shown in Figure 7 of Seah et al. [12]. These profiles may be converted to profiles as a function of depth allowing for the different sputtering yields of the FMOC and Irganox 1010. Measurements show that the yield is unchanged from that of Irganox 1010 until the FMOC content exceeds 80% [12]. We therefore recalibrate the depth scale on the basis of a relative yield rising linearly from 1 to 1.3 as the volume content of FMOC increases from 0.8 to 1.0 [12]. This may seem a little crude but, as we shall see in the analysis that follows, the interesting data occur in the region where the FMOC content is less than 80% and this small adjustment can be viewed as very fine tuning. Profiles for the normalized intensities as a function of the yield-corrected depth are shown in Figure 2(a) for secondary ions with *Ξ* < 0.2 which are expected to be closest to the true compositional profiles and in Figure 2(b) for the average of those eight profiles and a further eight with *Ξ* values up to the maximum *Ξ* value observed here. If *Ξ* were not known, it would be unclear exactly where the interface was and whether some of the profiles indicated compositional changes such as degradation or reaction with the next layer material. The depth resolutions shown are in the middle of the ranges given in the many studies reported by Seah et al. [23] and Shard et al. [24] and at the low end of the wider study by Shard et al. [30].

Note that the shifts at the two interfaces for different secondary ions are in different directions and to different extents. Thus, sputter rates determined directly from the intensity profiles would be wrongly lowered for the FMOC and raised for the Irganox 1010 to an extent governed by the *Ξ* values.

We can see that the profiles shift and that the shifts are worst as *Ξ* increases from zero. Ideally, we need a profile at *Ξ* = 0 but, in many cases, the analyst does not know the values of *Ξ*. Plots of the shifts as a function of *m*/*z* (not shown) give results that fill the space between 0 and 0.1 nm per *m*/*z* for the first interface and between 0 and −0.02 nm per *m*/*z* for the second interface so that low *m*/*z* ions are generally best although selected high *m*/*z* ions can also show small shifts. At *m*/*z* = 40, the combined shift can be 6 nm so, if *Ξ* is unknown, the use of a low mass ion may give the interface position uncertain to this level. To improve the accuracy of setting the interface positions, we now consider the effects of *Ξ*.

The profiles out of the FMOC layer at 100 nm (interface 1) are clearly broader than those into the FMOC at 200 nm (interface 2), despite the fact that the latter involves further sputtering. In studies of the delta layers in these materials to 300 nm depth [24, 29], the Ar GCIB sputtering depth resolution remains constant with depth and so this improvement reflects the material being sputtered. The positions of the profiles with higher *Ξ* values move inwards into the region of the Irganox 1010 from 100 to 200 nm. This effect is already observable in Seah et al. [12] with less detail. At interface 1, the profiles shift more than those at interface 2, with shifts of up to 8 and 4 nm, respectively, as *Ξ* increases to 0.8. At higher *Ξ* values, as can be seen in Figure 2(b), the shift can reach nearly 30 nm! Most of the changes in the intensity profile are in the Irganox 1010 region where the sputtering rate is constant and that greatly simplifies matters.

Results for Irganox 1010 secondary ions, which mainly show suppression (*Ξ* < 0), are broadly complementary to the FMOC data with the shifts at interface 1 also being to greater depths and those of interface 2 in the reverse direction. Again, interface 2 is about half the width of interface 1. Shard et al. [30] illustrate this for a few of the Irganox 1010 ions. Irganox 1010 data will be discussed no further in the present work.

The profiles at interface 2 at 200 nm are close to integral Gaussians but those at interface 1 at 100 nm clearly exhibit exponential tails of the type formed by integral Dowsett functions [1–3]. We shall return to this later, but using integral Gaussians is commonplace and, with their fewer independent variables, help to explain some of the observed behavior.

We shall consider the widths and positions of the profiles at each interface for all the ions to establish the underlying physics of the process so that the reader can see the magnitudes of the effects caused by the matrix terms. In most work, the matrix terms are unknown and so this also shows the magnitudes of errors where matrix terms are ignored.

### Profile Shifts, Ξ < 0.8, Integral Gaussian Model

**Figure 3:**
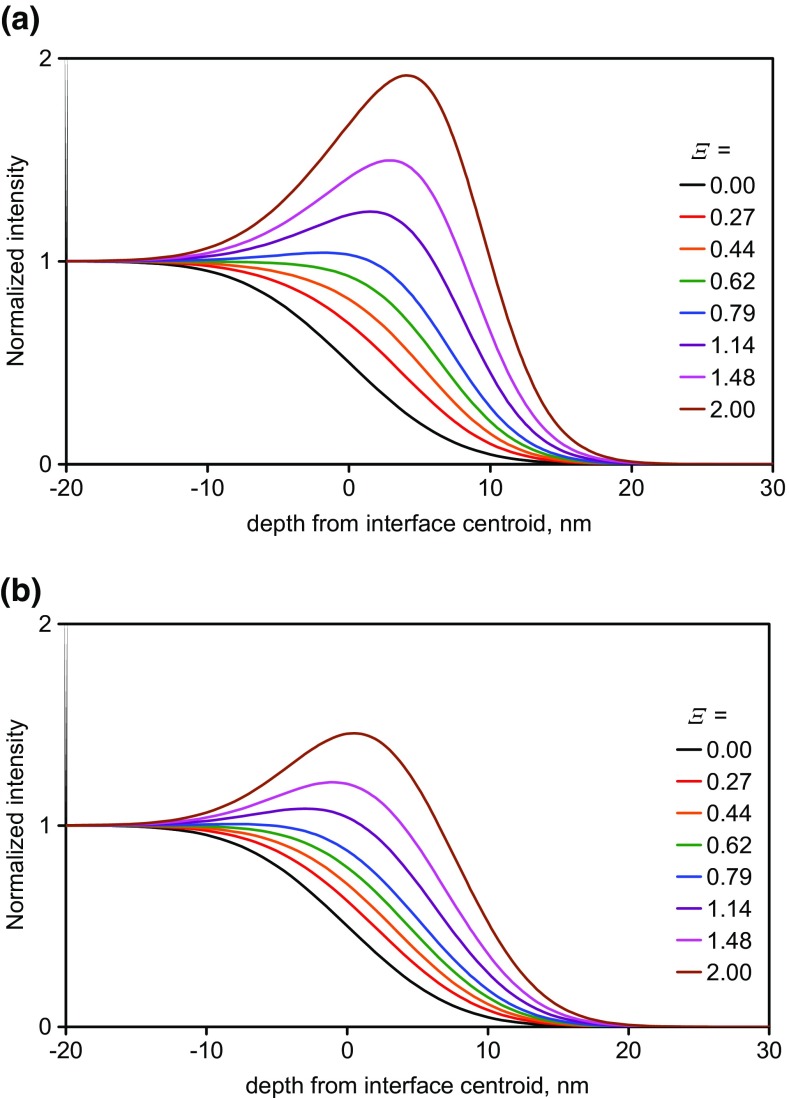
Emitted normalized intensities showing the effect of long wavelength roughening for a model integral Gaussian compositional profile for FMOC centered at 0 nm and with a measured standard deviation width, *σ*_M_, of 6 nm and in (**a**) with no long wavelength roughening and in (**b**) with 4.5-nm long wavelength roughening. The normalization is made by the ratio of the intensity measured to that for the pure material

The profiles in Figure 2(b) with *Ξ* < 0.8 exhibit no peak and are one starting point for a model to understand how intensities arise. If we model the interface composition by an integral Gaussian function with a width *σ*_c_ of 6 nm centred at 0 nm, the resulting intensity is shown in Figure 3(a) for many positive values of *Ξ*. In this model, the curves for the enhanced signal (as distinct from the composition) as a function of depth may also be described fairly accurately by an integral Gaussian provided that *Ξ* does not exceed 0.8. In this region of *Ξ*, the measured interface intensities shift to greater depths to an extent dependent on both the original width and *Ξ*. The shift is in the opposite direction for negative values of *Ξ* (the case for most of the Irganox 1010 secondary ions). A significant sharpening in the apparent depth resolution appears to occur, reaching a reduction of 40% at *Ξ* = 0.8 (and, similarly at *Ξ* = −0.8, not shown). Thus, the effective resolution is not necessarily that for the secondary ion with the best resolution if that ion has enhancement or suppression. Only those ions with values of *Ξ* close to zero may be taken to illustrate the true depth resolution being achieved in the profile. If *σ*_c_ is not 6 nm, then the abscissa in Figure 3(a) is simply scaled by *σ*_c_/6 where *σ*_c_ is in nanometres.

If *Ξ* exceeds 0.8, a peak appears in the intensity profile in the interface region since, as shown in Online Resource 1, Figure S.1, the intensity will exceed that of the pure material at some intermediate composition. Such a peak cannot be accurately fitted, directly, by the integral Gaussian without added terms. If *Ξ* exceeds 1.2, the peak is significant and may be used for fitting other effects as we shall discuss later. In Figure 3(a), it is assumed that the composition measured by the analytical ions is described by an integral Gaussian of width *σ*_c_; however, in practice, each secondary ion of a different mass is generated from a slightly different part of the analytical ion impact crater leading to small individual shifts in depth. Additionally, each of those analytical ion impact craters is at a slightly different depth due to the long wavelength roughness of the surface formed by the argon cluster sputtering [27, 29]. This longer wavelength roughness, *σ*_R_, must be combined with *σ*_c_ to give the measured roughness of the profile, *σ*_M_ for *Ξ* = 0. This measured roughness, *σ*_M_, is the roughening of the measured interface, i.e. its blurring, not the physical surface roughness at that moment in time which, since *σ*_c_ contains churning and mixing contributions, will generally be less than *σ*_R_. An example of the surface form in such sputtering is shown in Online Resource 1. Furthermore, the longer wavelength roughness, *σ*_R_, will also contain contributions from any unevenness in both the layer thickness and the dose over the whole of the crater area being analysed by the Bi_3_^+^ beam. Thus, *σ*_R_ may be significantly more than the physical local roughness formed by the sputtering. For the above Gaussians,

4 $$ {\sigma}_{\mathrm{M}}\kern0.36em ={\left({\sigma}_{\mathrm{c}}^2+{\sigma}_{\mathrm{R}}^2\right)}^{0.5} $$

The intensity is thus generated by enhancement at the surface of the sample with a compositional profile governed by *σ*_c_ and then that enhanced intensity profile is smoothed by being convolved with the longer wavelength roughness, of width *σ*_R_ as shown in Figure 3(b).

In Figure 3(b), *σ*_M_ is taken as 6 nm with *σ*_c_ = 4 nm so that *σ*_R_ is 4.5 nm. Both Figures 3(a) and (b) are for *σ*_M_ = 6 nm at *Ξ* = 0 but the enhancement effects in Figure 3(b) are reduced compared with Figure 3(a) by the long wavelength roughening. In the terminology of our study on delta layers [29], Figure 3(a) here is the result of a two-step approach, whereas Figure 3(b) is for a three-step approach that includes the long wavelength effects.

The values of *σ*_R_, *σ*_c_ and their ratio may all be dependent of both the Ar GCIB sputtering energy and cluster size as well as the Bi_3_^+^ analysis beam energy and any sample rotation used.

**Figure 4:**
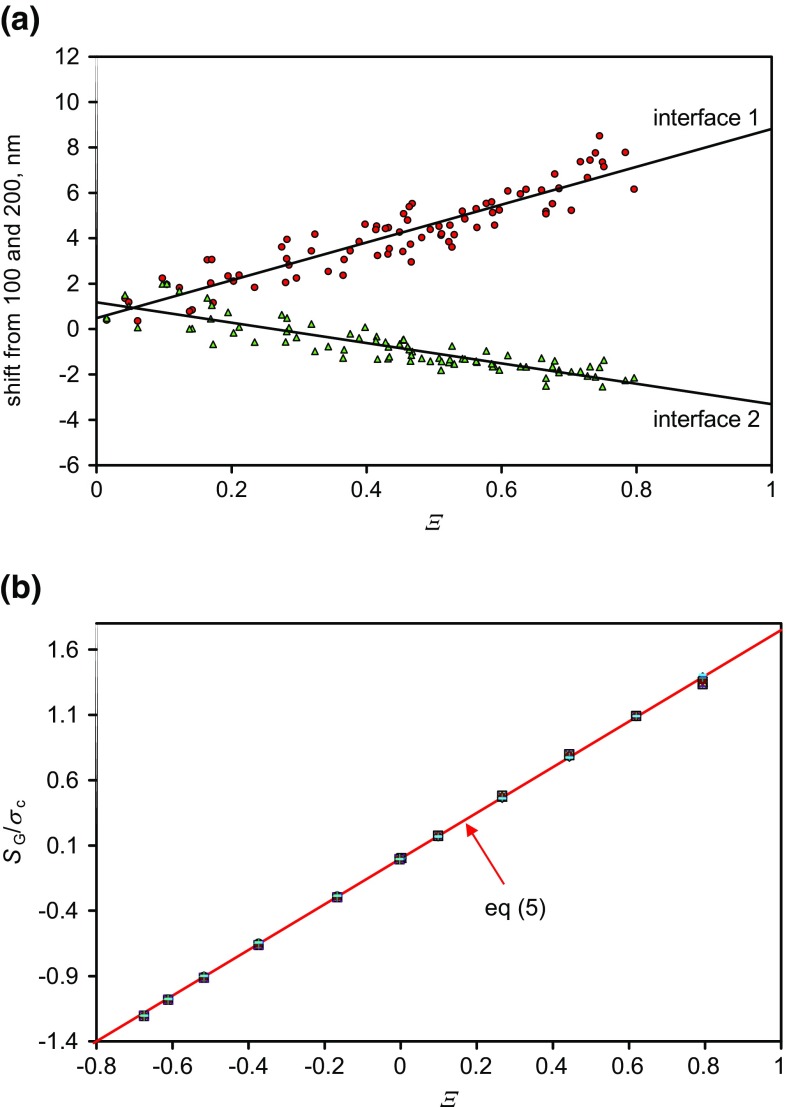
Shifts in the first and second interfaces for ions with *Ξ* < 0.8 deduced by directly fitting integral Gaussian functions, (**a**) experimental data (the shifts are in opposite directions as both move away from the FMOC layer), (**b**) calculations for the shifts for integral Gaussian compositional profiles for many values of both *σ*_c_ and *σ*_R_

The measured shifts in the two interfaces, by fitting integral Gaussians to the data with *Ξ* less than 0.8, are shown in Figure 4(a). These profiles shift over some 10 nm. The results are very similar to the shift calculated for the center of mass of the resolution function deduced by summing the raw normalized intensities as shown in Figure 2(a) over the depths from 50 to 150 nm and from 150 to 250 nm. The normalized shifts, *S*_G_, calculated from the integral Gaussian model compositions of Figure 3 are shown in Figure 4(b).

For secondary ions with *Ξ* < 0.8, Figure 4(a) shows the shift, *S*_G_, in the center of the fitted integral Gaussians for the intensities at the two interfaces. The ordinate origin is approximate since we have chosen to place the 100 and 200 nm positions approximately; however, it is the rate of shift that is important. From these data, the gradients of the data for the shifts per unit *Ξ* at interfaces 1 and 2 are 8.33 and −4.49 nm, respectively. The computed result for the model data, shown in Figure 4(b), gives a gradient that depends on the value of the standard deviation of the composition, *σ*_c_, at the interface such that:

5 $$ {S}_{\mathrm{G}}\kern0.36em =1.75\;{\sigma}_{\mathrm{c}}\;\varXi $$

The shift does not depend on *σ*_R_. Long wavelength Gaussian roughening will simply broaden the interface, but not shift it. From Eq. 5, we deduce from the shifts that *σ*_c1_ = 4.76 nm and *σ*_c2_ = 2.56 nm for the first and second interfaces, respectively. Here, we have assumed that *σ*_c_ is the same for all of the secondary ions at each of the interfaces and that the only changes arise from the enhancement effects. This is not quite true since each ion arises from a different region of the Bi_3_^+^ crater [27]. This may be the cause of the scatter about the lines in Figure 4(a). The standard deviations of the data about each solid line are 0.7 and 0.5 nm, respectively. It is likely that any individual result taken at a *Ξ* value of zero will be uncertain to that extent, whereas the true average positions, given by the intercepts of the straight lines, have smaller standard uncertainties due to the large number of measurements (84) in Figure 4(a). The standard uncertainty for interface 2 is 0.14 nm since the interface is close to an integrated exponential and Figure 4(b) predicts a straight line. However, the uncertainty for interface 1 is significantly worse since the expected line is not quite straight. Clearly, knowing *Ξ* has allowed us to establish the average position at *Ξ* = 0 to an uncertainty one to two orders of magnitude better than using a single profile of unknown *Ξ*.

The depth resolutions obtained in this study are in the range typically found for Ar GCIB profiling of organic materials [30] and illustrate normal practice. If by using lower energy sputtering or analysis ion beams or by sample rotation, *σ*_c_ is reduced, the shifts shown in Figure 4 are similarly reduced. Conversely, with poorer conditions, the shifts may be increased.

We next consider the resolutions at the interfaces from the secondary ion intensities such as those in Figure 2 for integral Gaussian resolution functions.

### Profile Widths, Ξ < 0.8, Integral Gaussian Model

**Figure 5:**
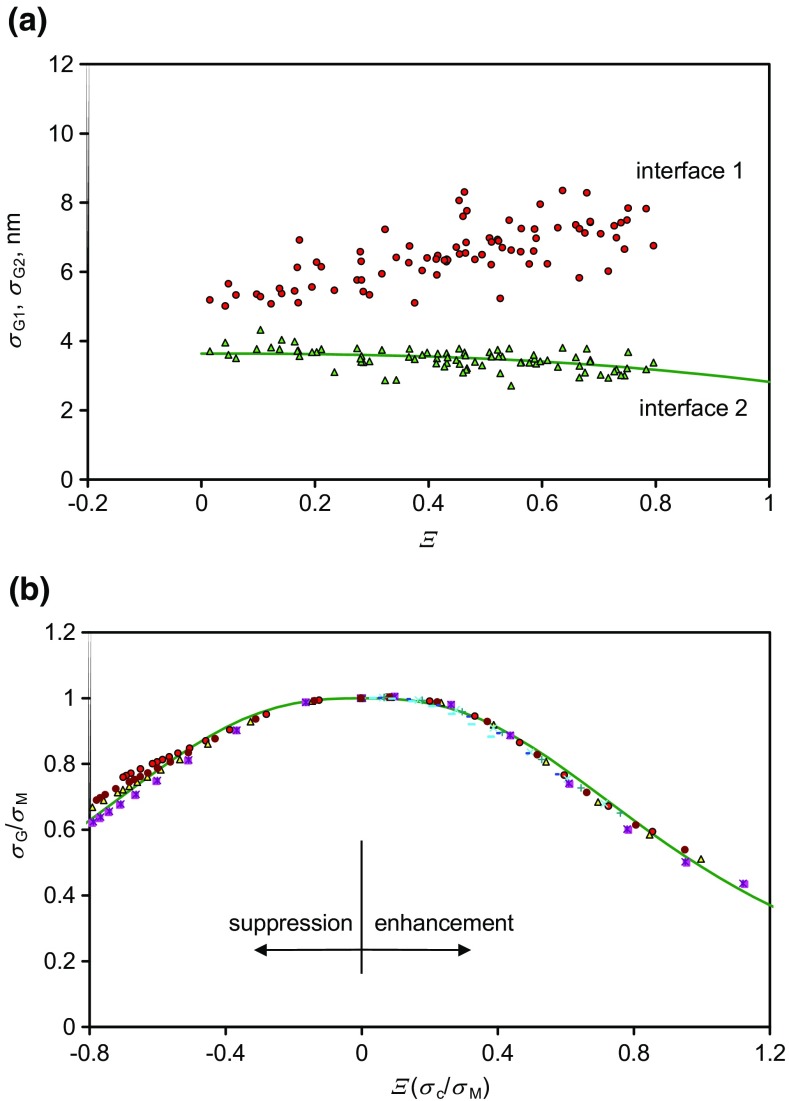
The widths as a function of *Ξ*, (**a**) experimental data (**b**) calculated fitted integral Gaussian width for enhanced model integral Gaussian profiles as a function of *Ξ* on normalized axes with a smooth curve to guide the eye. This smooth curve appears in (**a**) for interface 2

The measured interface widths, *σ*_G_*,* are shown in Figure 5(a) for the directly fitted integral Gaussian functions. We may expect the fitted resolutions to be poorer than *σ*_c_ since there will be a broadening of the intrinsic resolution sampled by the Bi_3_^+^ ions as a result of the longer wavelength roughness, *σ*_R_, generated by the argon cluster ion beam sputtering. However, this simple Gaussian theory always predicts a narrowing of the apparent resolution as *Ξ* increases (until at *Ξ* > 1.2, a peak appears in the profile and the integral Gaussian fitting is inappropriate). The analysis for the breadth of the fitted integral Gaussians is a little complex but, for various values of *σ*_c_, *σ*_R_ and *σ*_M_, is shown in Figure 5(b) on reduced axes. As the enhancement increases or reduces from zero, the non-linearity increases and the depth resolution appears to improve.

For the second interface, the apparent resolution improves from around 3.8 to 3.0 nm as *Ξ* reaches 0.8. The solid green curve in Figure 5(a) from the result in Figure 5(b) uses *σ*_M2_ = 3.64 nm and the value for *σ*_c2_ of 2.56 nm as determined earlier. Thus, *σ*_R_ = 2.59 nm and, as shown in the Online Resource 1, Figure S.2, atomic force microscope measurements indicate that the roughness, Sq of the surface form is significantly less than this, as noted above. This analysis predicts that the fitted integral Gaussian will never have a width greater than *σ*_M_ no matter what *σ*_c_ and *σ*_R_ values are used. This describes the second interface but does not the first. We shall return to this important issue at 4.5, later. However, it is clear that the data for the secondary ion with an intensity profile that looks best resolved will not give the best interface position, it will have a *Ξ* value around 0.8 and be significantly shifted.

### Intensities, Ξ > 1.2, Integral Gaussian Model

**Figure 6:**
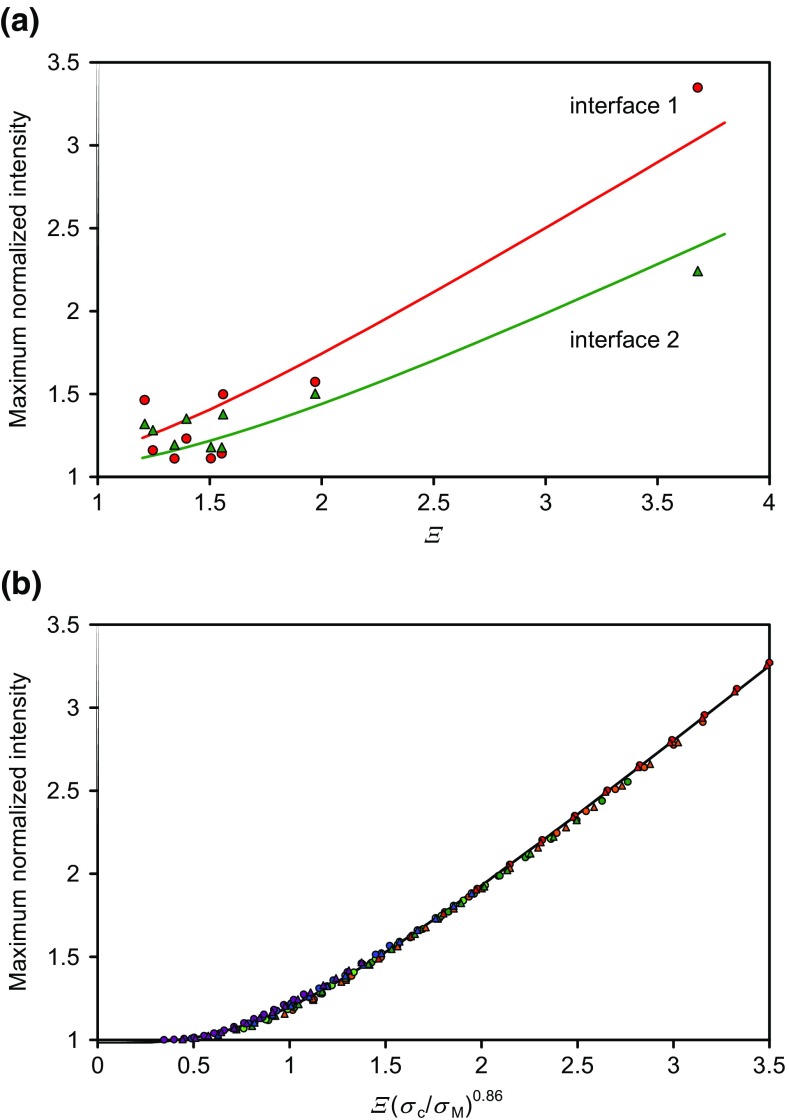
The maximum normalized intensities at the interface peaks, (**a**) experimental data for nine secondary ions with *Ξ* > 1.2 for the first (red) and second (green) interfaces as a function of *Ξ*, (**b**) calculations based on integral Gaussian interfaces for different values of *Ξ*(*σ*_c_/*σ*_M_)^0.86^. The smooth curves in (**a**) are the fitted smooth curve through the data shown in (**b**) for (*σ*_c1_/*σ*_M1_) = 0.90 and (*σ*_c2_/*σ*_M2_) = 0.70, respectively

We now consider the maximum intensities of the interface profile peaks seen in Figure 2(b) for *Ξ* greater than 1.2. There are nine secondary ions with *Ξ* > 1.2 as shown in Figure 6(a). The maximum intensities at the peaks for interface 1 are generally higher than those at interface 2, indicating a *smaller* effect of *σ*_R_. If *σ*_R_ is actually the same for both interfaces, the greater value of *σ*_M1_, for interface 1, compared with *σ*_M2_, would correlate with the higher intensity there. If *σ*_R_ was irrelevant, the maximum intensities associated with the two interfaces would be the same. For integral Gaussian compositional profiles, it is simple to calculate the maximum observed intensity for many values of *Ξ*, *σ*_c_ and *σ*_M_. This may be presented on a reduced plot as in Figure 6(b) showing how *Ξ* is effectively reduced as *σ*_c_/*σ*_M_ falls. If (*σ*_c2_/*σ*_M2_) is unity we get a description of the maximum normalized intensity seen in Online Resource 1, Figure S.1.

The ratio (*σ*_c2_/*σ*_M2_) = 0.70 for the second interface is shown by the green line in Figure 6(a) and is consistent for the apparent interface shift giving *σ*_c2_ = 2.56 nm and the width of the second interface giving *σ*_M2_ = 3.64 nm for *Ξ* < 0.8. This describes well the maximum intensities with *Ξ* > 1.2 shown in Figure 6(a). The first interface is not quite so well described.

**Figure 7:**
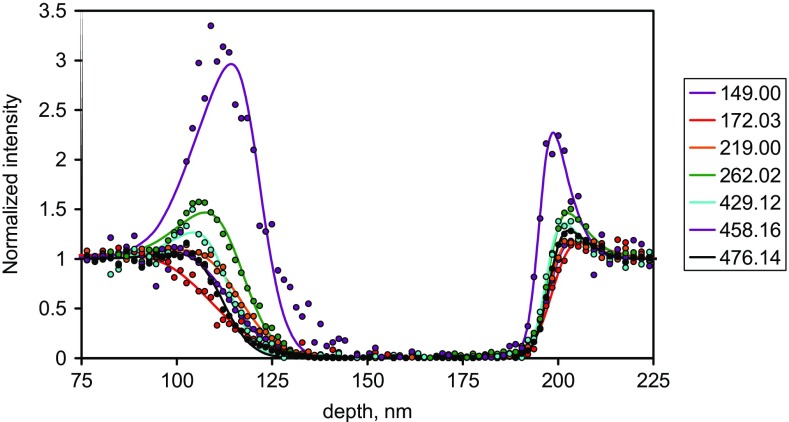
Normalized data and the fits for secondary ions with *Ξ* > 1.2 at the two interfaces using integral Gaussian functions to describe the composition with the mean positions, sigma values and *β* used as fitting values. The boxes to the right identify the secondary ion masses in *m*/*z*

We now fit the profiles for the data for *Ξ* > 1.2 where a distinct maximum appears in the profile. The fits using integral Gaussian functions to describe the interface, with the fitted mean positions, sigma and *β* values, are shown for seven of the nine secondary ions in Figure 7. The fits at the second interface are very good but those at the first interface are not so good. The peak intensity appears to be shifted too deep there. The ratio of the fitted *Ξ* value, *Ξ*_eff_, to *Ξ* for the nine secondary ions is 0.70 for the first interface and 0.77 for the second. For the strongest peak at *m*/*z* = 458, the values of *Ξ*_eff_/*Ξ* are 0.86 and 0.65, respectively, with *σ*_c1_ = 9.7 nm and *σ*_c2_ = 4.5 nm. The smaller values for the second interface arise since it is, overall, narrower. However, we shall see in the next section that the results for the first interface can be significantly improved.

The reason why the calculated shape for the first interface profile for *m*/*z* = 458 and other ions is poorer than for the second, seems to be the significant exponential decay contribution. The fitted compositional profiles so far have all been integral Gaussians whereas, when we have detailed profiles of delta layers, we use the more complex Dowsett function [1–3] which is the result of convolutions of a Gaussian with leading and with trailing exponential functions. We consider that next for all the FMOC secondary ions.

### Intensities, Ξ > 1.2, Integral Exponentially Modified Gaussian Model

In general, fits to describe a delta layer composition with Dowsett’s function [1–3] usually have a short rising exponential convolved with significant Gaussian and falling exponentials. We should integrate this delta shape to generate the compositional profile for a step edge. Instead of doing this numerically, an analytical solution is available. For the case where the short rising exponential may be ignored, this problem has long been solved [31] for lineshape and peak matching in other fields where the relevant functions are known as the exponentially modified Gaussian (EMG) distribution and the cumulative exponentially modified Gaussian distribution (i.e. the integral function). The composition depth profile *ϕ*(*x*) as a function of depth, *x*, for the first interface with its Gaussian part centered at depth *μ* with width *σ*, convolved with an exponential decay of length *λ*, is given by [31].

6 $$ 1-\phi (x)\kern0.36em =\Phi\;\left(u,0,v\right)-\exp \left\{-u+\frac{v^2}{2}+\ln \left[\Phi \left(u,{v}^2,v\right)\right]\right\} $$

where Φ(*x*,*μ*,*σ*) is the integral Gaussian, *u* = (*x* − *μ*)/*λ* and *v* = *σ*/*λ.* Unlike the case for the symmetrical Gaussian, the centroid for this function is at *μ* + *λ* instead of the Gaussian center, *μ*.

**Figure 8:**
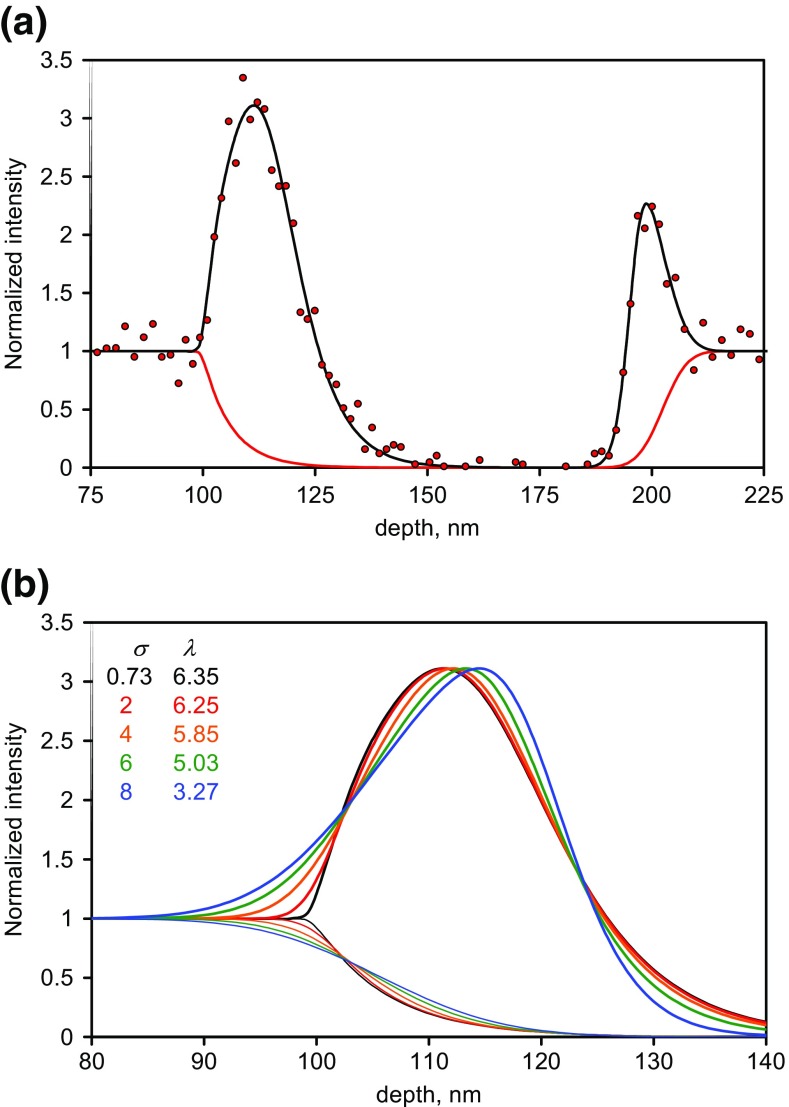
Intensity and compositional profiles calculated for the integral EMG model, (**a**) for *m*/*z* = 458 secondary ions with interface 1, *μ* = 99.6 nm, *λ* = 6.4 nm, *σ* = 0.7 nm, *Ξ* = 3.32 and for the second interface, *μ* = 203.3 nm, *λ* = 1.0 nm, *σ* = 4.3 nm, *Ξ* = 2.4 together with the experimental data, (**b**) curves for interface 1 with the same overall intensity and centroid for the composition depth profile but *σ* is increased from 0.73 to 8 with *λ* declining so as to maintain the same overall signal intensity integrated over depth

In Figure 8(a), the fits using this function to the *m*/*z* = 458 data are shown and, for the first interface, the function is a significantly better description than the simple integral Gaussian. For interface 1, *μ* = 99.6 nm, *λ* = 6.4 nm, *σ* = 0.7 nm and *Ξ*_eff_ = 3.32 so that *Ξ*_eff_/*Ξ* = 0.90. For interface 2, in Eq. 6, the left hand part, 1 − *ϕ*(*x*), is replaced by *ϕ*(*x*), *μ* = 203.3 nm, *λ* = 1.0 nm, *σ* = 4.3 nm and *Ξ*_eff_ = 2.4 so that *Ξ*_eff_/*Ξ* = 0.65. These *Ξ*_eff_/*Ξ* values at the second interface are similar to those found using the integral Gaussian model to describe the composition. The first interface exhibits large *λ* and small *σ* values whereas at the second interface *λ* is relatively weak. This reversal explains why the integral Gaussian has already described the essential behavior of interface 2.

Observation of the delta layer profiles for Irganox 1010 in FMOC and FMOC in Irganox 1010 are shown towards the right in Figure 7 of Seah et al. [12]. The Irganox 1010 delta profiles in FMOC may all be fitted with symmetrical Gaussian functions very precisely, whereas as detailed by Seah et al. [29], the delta profiles for FMOC in Irganox 1010 are very asymmetric with rapid rising edges (*λ*_u_ = 1.5 nm) and a slower exponential tail (*λ*_d_ = 5.4 nm). If these shapes are determined by the sputtering properties in the relevant matrices, then sputtering in, or into, FMOC, should be symmetrical (interface 2) and into Irganox 1010 should be asymmetrical (interface 1)—as, indeed, we do observe here. Asymmetry is also seen for delta layers of Irganox 3114 in Irganox 1010 [8].

Figure 8(b) shows how the profiles will change as the parameters are shifted from a high *λ* and low *σ* combination to a low *λ* and high *σ* combination at a constant overall signal intensity and constant compositional profile centroid. The observed intensity peak can be seen to shift to smaller depths relative to the centroid of the composition as the exponential part dominates.

**Figure 9:**
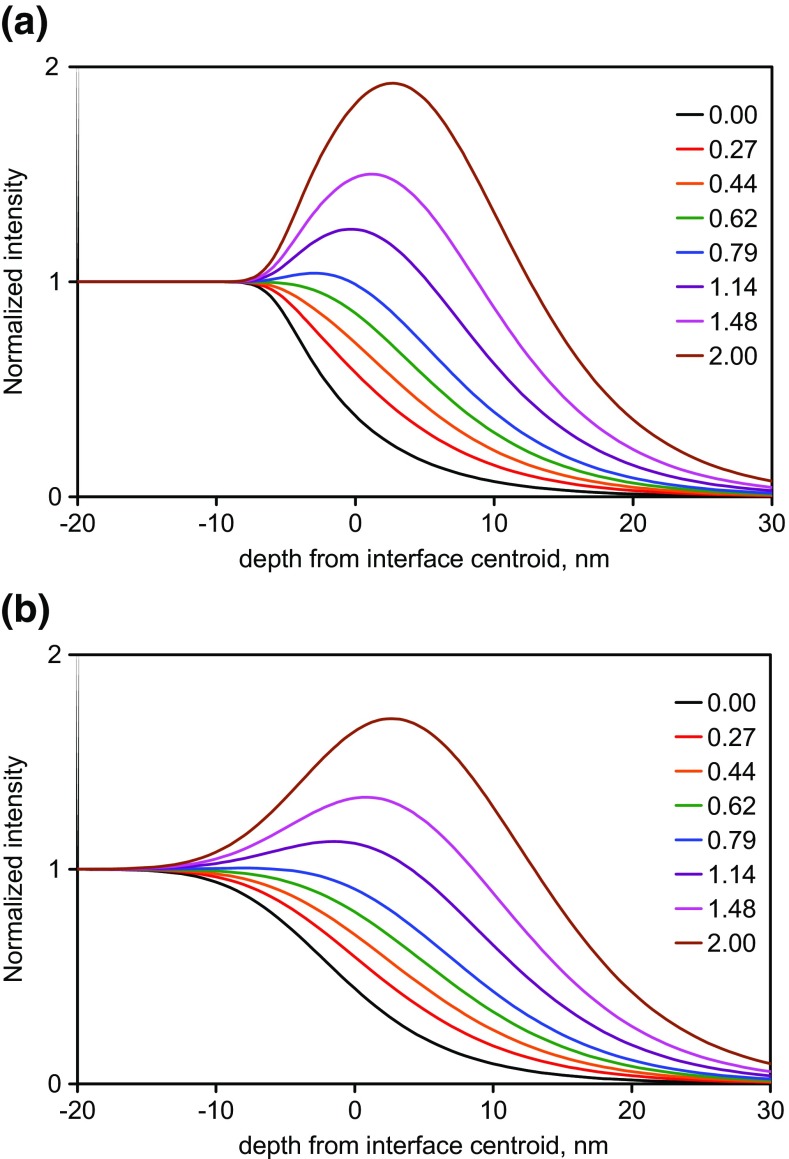
Emitted intensities for an integral EMG compositional profile for FMOC with *λ* = 6 nm, *σ* = 1 nm and *μ* = −6 nm and in (**a**) with no long wavelength roughening and in (**b**) with 4.5 nm roughening. The values of *Ξ* used are shown to the right

We now need to see what a similar treatment to that for the symmetrical integral Gaussian tells us about the first interface. In Figure 9(a), the analogue to Figure 3, we show the calculated intensity profiles for a compositional profile with *λ* = 6 nm, *σ* = 1 nm and *μ* = 0 nm for *Ξ* values in the range 0 to 2. The compositional centroid is at 6 nm. In Figure 9(b), the same profiles have then been convolved with the Gaussian roughening with standard deviation 4.5 nm. Whilst Figures 3(a) and 9(a) are quite distinct, by the time the long wavelength roughening is added, the results become more similar. Indeed, it now becomes uncertain to fit the lower *Ξ* functions in Figure 9(b) with the original integral EMG to deduce a *Ξ*_eff_ since the increased number of variables leads to excellent fits for various combinations of the values of the now correlated variables. In particular, even with a known *Ξ*_eff_, a smaller *σ* and larger *λ* give small changes to the profile but importantly cause a shift to the centroid over some 2 nm. There is, of course, the added issue that the long wavelength roughness itself may need to be described by an integral EMG at this particular interface, adding further uncertainty.

### Interface 1, Integral Exponentially Modified Gaussian Model

**Figure 10:**
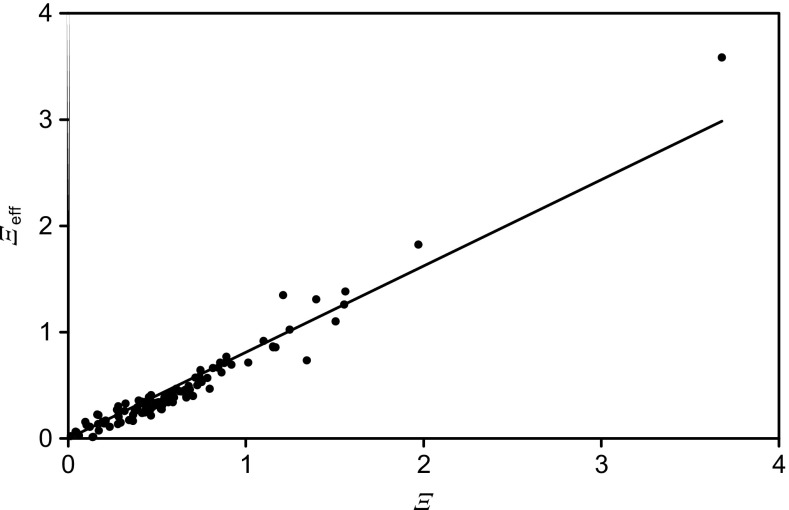
The value of *Ξ*_eff_/*Ξ* for interface 1 when *σ* is set at 1.1 nm and *λ* at 5.9 nm. The line shows *Ξ*_eff_/*Ξ* = 0.8

We now analyse all of the data at interface 1 using the integrated EMG compositional profiles. The small term *σ* for *v* in Eq. 6 varies around 1.1 nm and since this is small, it is fixed at that value for all secondary ions. Next, it is found that *λ* averages 5.9 nm and so, if that value is fixed for all secondary ions and the data are fitted, we may deduce the compositional centroid and the dependence of *Ξ*_eff_ on *Ξ*. The latter is shown in Figure 10 where the solid line is the regression fit to all the data showing *Ξ*_eff_/*Ξ* = 0.8. This result, and the overall widths for the different secondary ions, is the same as found earlier for FMOC delta layers in Irganox 1010 [29]. As noted earlier, sputtering into Irganox 1010 at interface 1 is similar to sputtering through Irganox 1010 for the delta layers.

Setting *Ξ*_eff_/*Ξ* at 0.8 and *σ* at 1.1 nm allows the compositional centroid position to be deduced and now it is effectively constant although still scattered about a 2-nm region. The centroid position uncertainties are greater at the higher *Ξ* values and so we simply average the positions derived, after allowing for *Ξ*, for the 13 ions with *Ξ* < 0.25. This shows an uncertainty of 0.19 nm in the value of this first interface centroid. This is a similar uncertainty to that found using the simpler integral Gaussians and ignoring the long wavelength roughness. However, if an exponential decay is fitted with an integral Gaussian, the centroid derived is at too small a depth. For the values used here, the error is around 1 nm. Similarly, if the integral EMG decaying profile is fitted with an integral Gaussian, the centroids will also be systematically at too small a depth. Thus, the average centroid for the 13 ions above for interface 1 is 1.1 nm deeper than that found using fitting integral Gaussians to the measured data and extrapolating with a linear function to *Ξ* = 0 as in Figure 4. If the integrated EMG profile is applied directly without involving the enhancement effect, we see a similar apparent dependence on *Ξ* as observed using the integral Gaussians in Figure 4(a). The data show a slight curvature with *Ξ* so a fit is made using a quadratic function. This extrapolates to *Ξ* = 0 at 0.07 nm lower value than the average compositional centroid after correcting for *Ξ*. This is a factor of 14 times closer to the average composition centroid than that derived by fitting integrated Gaussians to the interface 1 data. Importantly, also, there is now the increase in width shown in Figure 5(a) for directly fitting the data with the integral Gaussian.

The data from interface 1 are therefore consistent with an integrated EMG compositional profile with the centroid of the secondary ion depths scattered over 2 nm and which then provides an enhanced intensity that is finally convolved with a long wavelength roughness contribution that is a smaller fraction of the total width than for the narrower interface 2. The data are insufficiently precise to determine if this broadening should also be described by an integrated EMG profile although that seems likely.

The analysis for interface 2 show low values for *λ* and are not significantly changed from the results for the analysis using integrated Gaussians. The position of the interface deduced from the compositional profiles for interface 1 and interface 2 for different secondary ions could be anticorrelated if there were residual unknown effects of *Ξ* but would be correlated if the depth of the average source of the emitted secondary ion was similar in craters in FMOC and in Irganox 1010. Analysis shows that they are correlated. The secondary ions that rise earlier at interface 1 then fall earlier at interface 2. A shift of 1 nm to greater depths at interface 2 is equivalent to a shift of 2 nm in the same direction at interface 1. This is not unexpected since the general broadening at interface 1 is twice that at interface 2. This confirms the importance of the analytical ion crater. The standard deviation of the distribution of the data about the position of interface 2 in Figure 4(a) is 0.48 nm. That for interface 1 is 0.71 nm so that, if the two were uncorrelated, the standard deviation of the differences in interface position by which one determined the Irganox 1010 layer thickness would be 0.86 nm. The correlation in the shifts reduces that value to a measured value of 0.61 nm. The scatter in the separations of the two interfaces is significantly less than the scatter for just interface 1.

In all of the above, we have used and assumed an approximate sputtering yield for the materials taken from earlier work. In practice, the yield may not be known or the interfaces may be required to evaluate that sputtering yield. Then, more correctly, the abscissa in the plots will represent the sputtering ion dose. We have used depth in the present work so that the extents of the effects, in nanometres, are clear. As a function of dose, the positions of the two interfaces are thus given by the profile fits extrapolated to *Ξ* = 0. The true volume sputtering yields are given by the true thicknesses divided by these dose differences.

## Conclusions

Measurements are interpreted of the SIMS profiles at the FMOC to Irganox 1010 and Irganox 1010 to FMOC interfaces for many different secondary ions. These lead to seven important conclusions, applicable to all organic profiles:

- The precise shape and position of the intensity profiles is strongly governed by matrix effects but the effects of long wavelength roughening weaken that effect by a small amount dependent on the relevant amplitudes of the analytical ion crater and the long wavelength roughness. This causes the relative positions of the interfaces from the intensity profiles to vary over 10 nm.
- The compositional profiles, after removal of matrix effects, are described by a simple integrated Gaussian at the Irganox 1010 to FMOC interface but by an integrated exponentially modified Gaussian at the converse interface.
- If traditional integrated Gaussian functions are used instead of exponentially modified Gaussians, the apparent centroid of the interface may be significantly in error even at *Ξ* = 0. If *Ξ* is not measured, the interface position may be up to 8 nm in error.
- The interface resolution measured for interfaces with *Ξ* above or below zero may appear to be sharper than the true resolution and this may lead analysts to select secondary ions with *Ξ* values around 0.8 as the “best” ions but with significant, hidden, profile shifts.
- The deduced compositional profiles are consistent at each interface but their precise positions for different secondary ions vary over some 2 nm. A correlation is found with a 1-nm shift in depth at interface 2 reflected by a 2-nm shift at interface 1. This indicates that the secondary ions may each arise from different average depths in the crater formed by the analytical ion.
- To deduce interface positions accurately, profiles from several secondary ions with *Ξ* values below 0.1 are recommended or the position must be deduced at *Ξ* = 0 by extrapolation from the effective position for ions with *Ξ* < 0.25, if possible, and certainly <0.8, as a function of *Ξ*. Here, with 84 results, the standard uncertainties of the positions are 0.19 and 0.14 nm, respectively.
- The extent of the effects reported here may change with experimental operating conditions but, in general, any choice such as low beam energies or sample rotation to improve the depth resolution will reduce the interface shifts observed and vice versa.

## Electronic supplementary material

ESM 1 (PDF 297 kb)
