# Supplementary material for: SIMS of Organic Materials—Interface Location in Argon Gas Cluster Depth Profiles Using Negative Secondary Ions
Source: J Am Soc Mass Spectrom. 2018 Feb 21;29(4):774–85. doi: 10.1007/s13361-018-1905-2 (PMC5889422; doi:10.1007/s13361-018-1905-2)
Supplement: Supplementary file 1 — (PDF 297 kb) [file 13361_2018_1905_MOESM1_ESM.pdf]

Online Resource 1

SIMS of Organic Materials – Interface Location in Argon Gas Cluster Depth Profiles Using Negative Secondary Ions

R. Havelund[1], M. P. Seah[1], M. Tiddia[2] and I. S. Gilmore[1]
[1] *National Physical Laboratory, Teddington, Middlesex TW11 0LW, United Kingdom*
[2] *Universita degli Studi di Cagliari, Dipartimento di Fisica S. P. Monserrato - Sestu Km 0.700, 09042 Monserrato CA, Italy*

S.1 Determining Matrix Parameters

To deduce the matrix parameters, the measured intensities for each secondary ion are normalized by the intensity from the pure material and equations (1) and (2) are applied with the constraint relating α to β. From this fitting, as shown in examples in Figure S.1, the Ξ value and the intensity as a function of ϕ_A may be determined. This quality of fitting is typical of all secondary ions in this study.

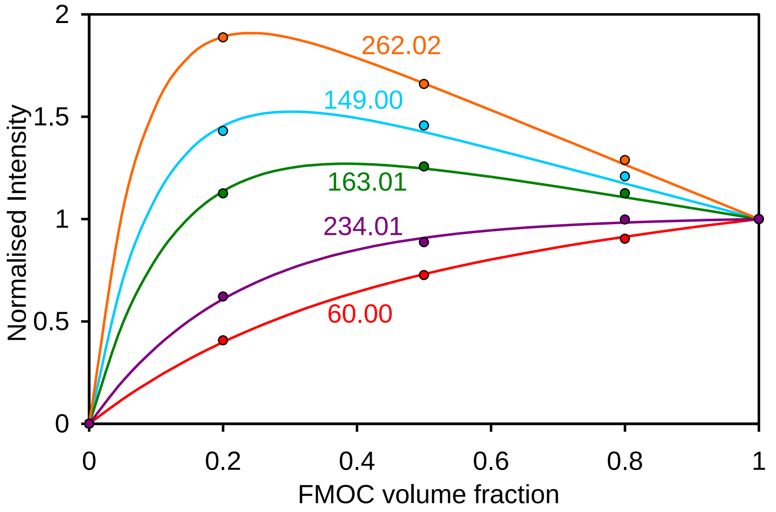

Figure S.1. Normalized intensities for five secondary ions from the uniform mixtures of FMOC and Irganox 1010 with $0 < \Xi < 2$ with fitted curves using equation (1) with $\alpha = [1 - \exp(-\beta)]P + Q\beta$. The Ξ values are, from the top, 1.97, 1.51, 1.17, 0.59 and 0.32.

S.2 Measuring the Surface Form Resulting from Argon Cluster Sputtering.

An example study of the surface form of Irganox 1010 by atomic force microscopy (AFM) after argon cluster sputtering is shown below. An Asylum Research MFP-3D Classic AFM was used with an OPUS 240 AC-PP AFM tip in the non-contacting mode. The image is for 256 by 256 pixels with a field of view of 10 µm by 10 µm. The image is after levelling, but no filtering, and the root mean square area surface roughness, given by Sq, is 1.6 nm. In a second crater, Sq is measured as 1.7 nm. The AFM data are not recorded in situ during the profiling but in air after terminating the sputtering. It is known that such organic surfaces can change their surface form significantly overnight and so these measurements are made within 30 minutes of the cessation of the sputtering. The Bi_3^+ analytical data in the depth profiles are recorded for many

individual points on such surfaces and those data are thus averaged over the respective depths in the profile to give the measured profiles. This, and other, long wavelength terms contribute to σ_R and reduce the effect of Ξ slightly, as discussed in the text. The surface roughness is thus an important, small, corrective factor in determining the measured profiles for individual secondary ions.

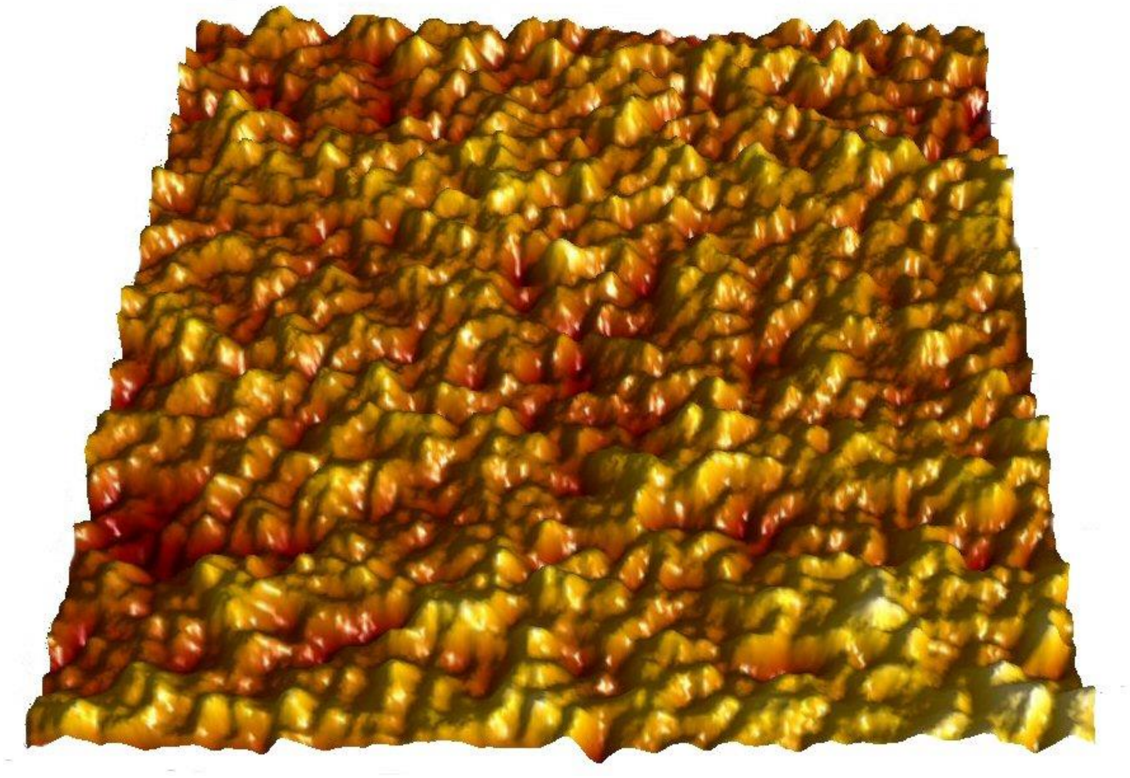

Figure S.2. 10 μm by 10μm 3D AFM image of the surface of Irganox 1010 after sputtering with argon cluster ions to 100 nm depth showing the surface form. The surface has been levelled but not filtered. The area roughness, S_q, is 1.6 nm. Note that, as usual, the height scale is enhanced by 100 times with respect to the lateral scales for presentation.
